# A long-surviving patient with advanced esophageal basaloid squamous cell carcinoma treated only with radiotherapy: case report and literature review

**DOI:** 10.1186/s12876-017-0714-6

**Published:** 2017-12-08

**Authors:** Toshiya Maebayashi, Naoya Ishibashi, Takuya Aizawa, Masakuni Sakaguchi, Homma Taku, Moritaka Ohhara, Toshirou Takimoto, Yoshiaki Tanaka

**Affiliations:** 1 Department of Radiology, Nihon University School of Medicine, 30-1 Oyaguchi Kami-cho, Itabashi-ku, Tokyo, 173-8610 Japan; 2 Department of Human Pathology, Division of Pathology and Microbiology, Nihion University School of Medicine, Itabashi-ku, Tokyo, 173-8610 Japan; 3 Department of Digestive Surgery, Kasukabe Medical Center, Kasukabe, Saitama, 344-8588 Japan; 4 Department of Pathology, Kasukabe Medical Center, Kasukabe, Saitama, 344-8588 Japan; 5 Department of Radiation Oncology, Kawasaki Saiwai Hospital, Kawasaki, Kanagawa 212-0014 Japan

**Keywords:** Esophageal basaloid squamous cell carcinoma, Radiation therapy

## Abstract

**Background:**

Esophageal basaloid squamous cell carcinoma (EBSCC) is a rare malignant disease. Advanced EBSCC (AEBSCC) has a poorer prognosis than the more common esophageal squamous cell carcinoma, but no treatment policy has yet been established. This is the first reported case with AEBSCC treated only with radiotherapy. Thus, our long-surviving patient merits consideration. We therefore reviewed cases with the same stage of AEBSCC for further investigation.

**Case presentation:**

An 85-year-old man with a chief complaint of difficulty swallowing foods was diagnosed with AEBSCC, cT3N1M0, stage III, by thorough examination. The basaloid carcinoma extended from the upper thoracic esophagus to the middle thoracic esophagus based on imaging studies, endoscopy and biopsy.

Morphologically, the tumor was an elevated ulcerative area. We conducted radiotherapy to relieve symptoms, as the patient and his family refused aggressive treatment. He has remained alive without recurrence for 2 years, to date, after completing radiotherapy.

**Conclusions:**

Basaloid carcinoma might be highly sensitive to radiotherapy. Thus, radiotherapy for local control might be beneficial for elderly patients with complications and those refusing aggressive treatment.

## Background

Wain et al. reported basaloid carcinoma for the first time among patients with head and neck cancers [1]. It is a rare histological form of esophageal cancer and reportedly accounts for 0.1% of cases with esophageal cancers [2, 3]. Advanced esophageal basaloid squamous cell carcinoma (AEBSCC) has a poorer prognosis than the more common esophageal squamous cell carcinoma (ESCC), but no treatment policy has yet been established [4].

According to our literature search, this is the first reported case with AEBSCC treated only with radiotherapy. Basaloid carcinoma might be highly sensitive to radiotherapy. Thus, radiotherapy for local control might be beneficial for elderly patients with complications and those refusing aggressive treatment. We evaluated 10 AEBSCC patients at the same disease stage for which detailed descriptions were available (Table 1).

**Table 1:** Clinical characteristics of 10 Japanese cases with stage III esophageal basaloid squamous cell carcinoma: site, morphology, metastasis, survival period, current status and treatment

Site	Morphology	Metastasis	Survival period (months)	Current status	Treatment
Upper and middle	Type 1 (Erosive elevation)	Bone	13	Dead	Surgery alone
Lower	Type 2	Lung, liver, lymph nodes	8	Dead	Chemoradiotherapy after surgery
Upper	Type 2	Lung, brain	22	Dead	Chemotherapy after surgery
Lower	Type 3	Liver	10	Dead	Surgery alone
Lower	Type 1	Liver	36	Alive	1. Chemotherapy after surgery 2. Chemotherapy after liver metastasis resection
Middle	Type 1	Liver, lymph nodes	9	Dead	Surgery after pre-operative chemotherapy
Lower	Type 1	Liver	27	Alive	1. Surgery 2. Surgery after hepatic arterial injection chemotherapy for liver metastasis
Middle	Type 2	Liver, lymph nodes	10	Dead	Chemotherapy after surgery
None	None	Mediastinal lymph modes, solitary lung	61	Alive	1. Surgery 2. Mediastinal lymph node radiation 3. Surgery after chemotherapy for lung metastasis
Upper	Type 3	None	25	Alive	Radiotherapy alone

## Case presentation

**Fig. 1:**
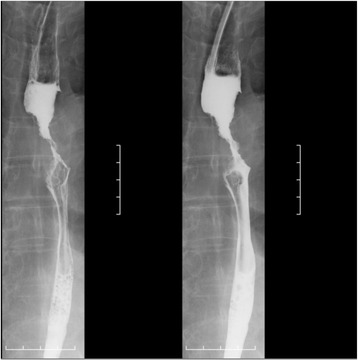
Esophagography: Extensive narrowing is seen on the oral side from the carina, and mild extension is present in the esophagus on the oral side. Passage of the contrast medium is possible and there are no fistulas in the carina

**Fig. 2:**
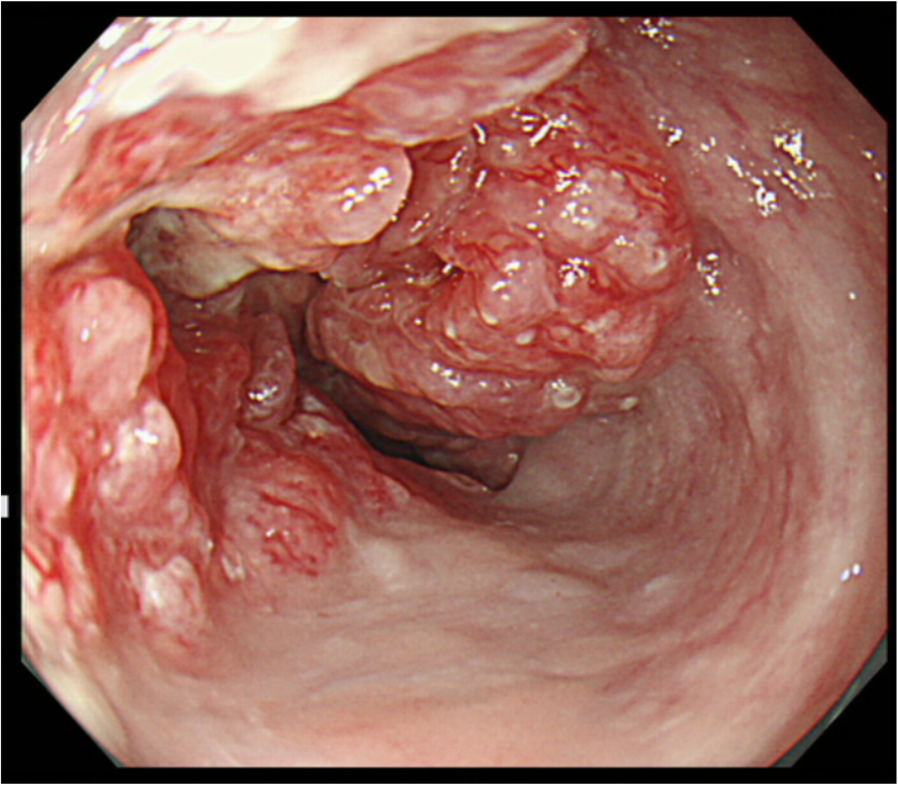
Upper gastrointestinal endoscopy: Macroscopic type 3 advanced esophageal cancer, which appears to be nearly circumferential, can be seen 22 cm from the gums. The tumor was speculated to have developed and then extended into the submucosal layer

**Fig. 3:**
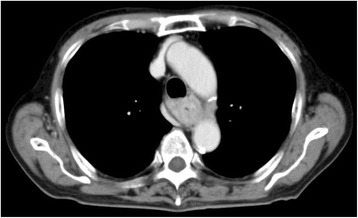
Computed tomography of the chest: An esophageal tumor, which appears to compress the membranous portion of the trachea, is considered to be indicative of advanced esophageal cancer as an *en bloc* mass with lymph node metastasis

**Fig. 4:**
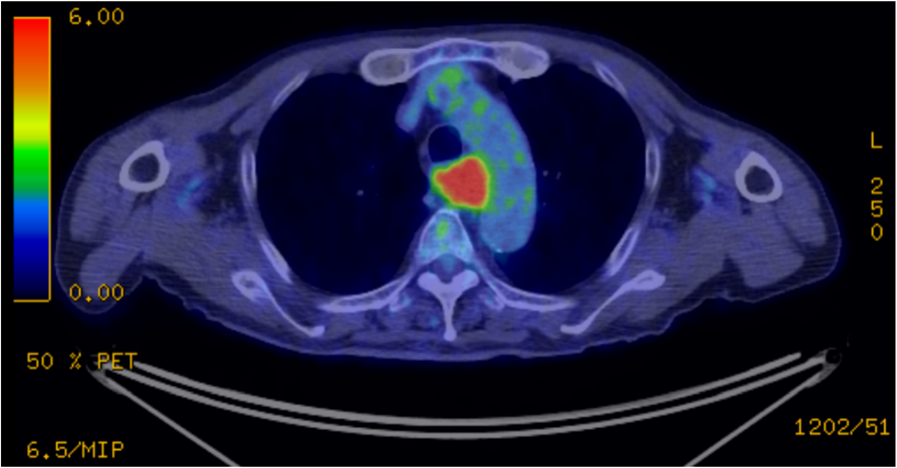
Positron emission tomography–computed tomography: There is radionuclide accumulation in the portion where the *en bloc* mass forms an esophageal tumor compressing the membranous portion of the trachea and lymph node metastasis is present, but there is no evidence of distant metastasis

**Fig. 5:**
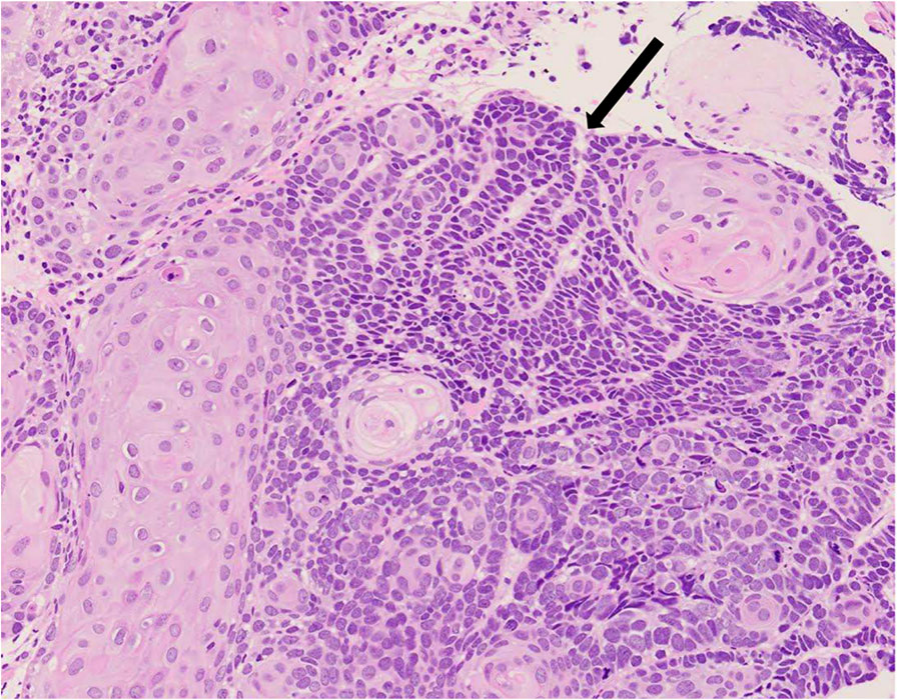
Biopsy histopathological image (Hematoxylin-Eosin staining: 20 X magnification): Small and spindle-shaped tumor cells with scanty cytoplasm are arranged in cords, forming a tumor nest similar to basal cells with no keratin pattern formation (narrow). Proliferation of atypical squamous epithelium is present around the nest and there are also components of squamous cell carcinoma

**Fig. 6:**
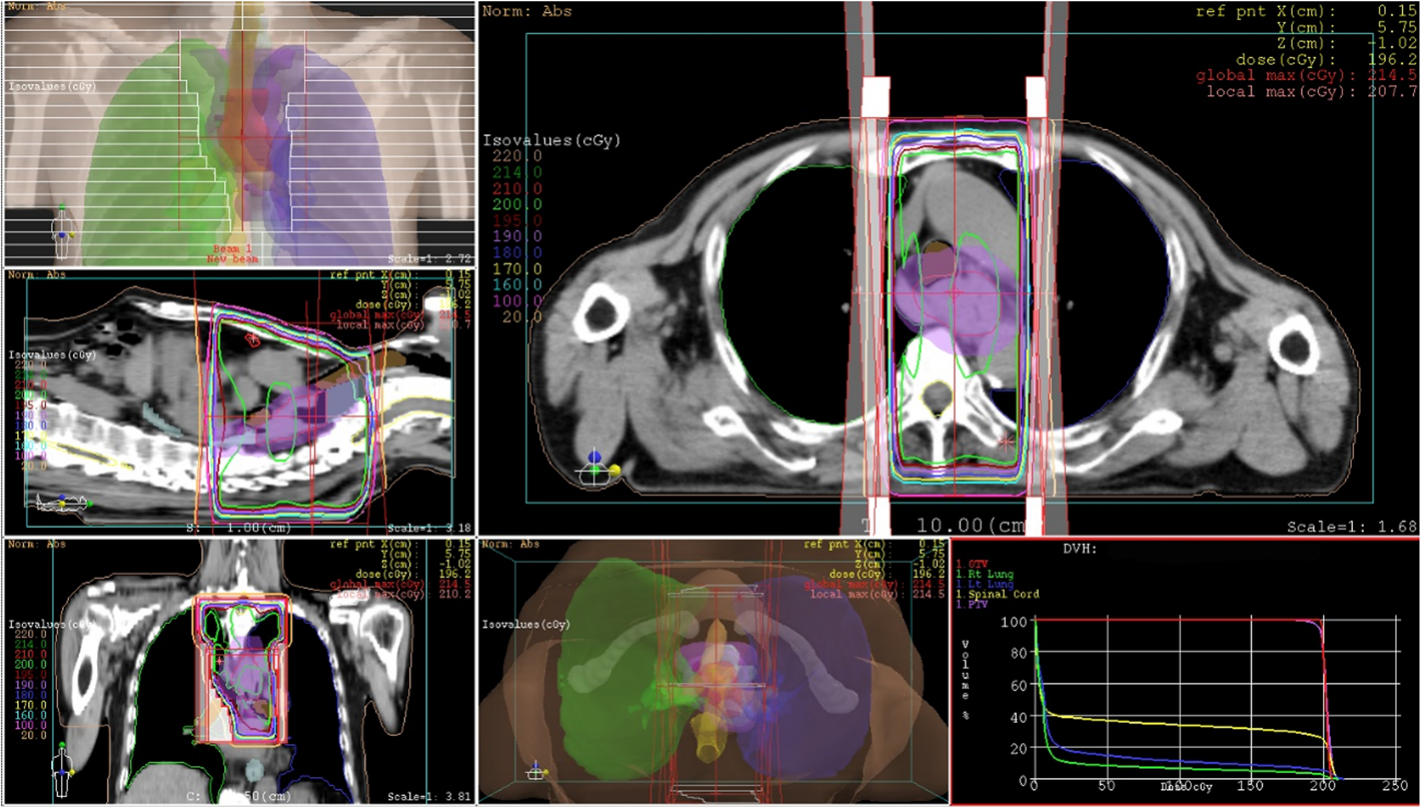
The irradiated fields and dose distributions for advanced esophageal basaloid squamous cell carcinoma irradiation using 10MV X-rays. He was prescribed a dose of 60 Gy in 2-Gy fractions, allocated as 40 Gy to the regional field and 20 Gy to the boost field. (holding the spinal cord dose below 40Gy)

**Fig. 7:**
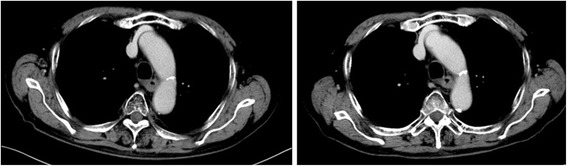
Computed tomographic images obtained 16 (right figure) and 25 months (left figure) after completion of radiotherapy for esophageal basaloid squamous cell. There has been no evidence of recurrence or metastasis since the initiation of this treatment

An 85-year-old man with a 1-month history of difficulty swallowing foods presented to our department and was diagnosed with AEBSCC, cT3N1M0, stage III, by thorough examination (Figs. 1, 2, 3, 4 and 5). The basaloid carcinoma extended from the upper thoracic esophagus to the middle thoracic esophagus based on imaging studies (Figs. 1, 3 and 4), endoscopy (Fig. 2), positron emission tomography–computed tomography (Fig. 4) and biopsy (Fig. 5). Morphologically, the tumor was an elevated ulcerative area. Furthermore, the tumor was found to have spread into the submucosa (Figs. 1 and 3). Immunohistochemical staining showed the tumor to be negative for p16. The patient had been diagnosed with prostate cancer 10 years earlier and had received hormone therapy for 5 years. There had been no recurrence of the prostate cancer. His medical history also included pulmonary tuberculosis and spinal stenosis. He smoked 20 cigarettes per day for the prior 12 years and drank 2 *go* (approximately 361 mL) of alcohol daily. We initially recommended surgery as aggressive treatment because his general condition was good and the prognosis of AEBSCC is poor. However, he refused aggressive treatments including chemotherapy. We thus administered radiotherapy for symptom relief. The radiation field ranged from the supraclavicular lymph node region to the entire esophagus, and radiation was delivered at a dose of 60 Gy in 2-Gy fractions (Fig. 6), allocated as 40 Gy to the regional field and 20 Gy to the boost field. To date, approximately 2 years have passed since radiotherapy completion. For follow-up of this patient with AEBSCC after radiation therapy, we obtained a detailed history and performed a full physical examination, computed tomography and upper gastrointestinal endoscopy every 3–6 months. The disease course has been good with neither recurrence nor metastasis and there were no adverse effects related to radiation therapy (Fig. 7). There were no late adverse events related to radiation therapy.

## Discussion and conclusions

Esophageal squamous cell carcinoma (ESCC) is the predominant form of esophageal cancer in Japan. Squamous-cell tumors comprise 98% of malignancies in the upper and middle third of the esophagus [5, 6].

Esophageal basaloid squamous cell carcinoma (EBSCC) is a rare histological form of esophageal cancer and reportedly accounts for 0.1% of cases with such cancers [2, 3]. Basaloid squamous cell carcinoma (BSCC) is a high-grade variant of squamous cell carcinoma of the head and neck [7]. Esophageal basaloid carcinoma is derived from esophageal epithelial basal cells or undifferentiated cells with similar multipotential features [8]. Therefore, it is considered to be difficult to identify this type of cancer by biopsy [9] and some reports have indicated that it constitutes 11.3% of esophageal tumors [10].

EBSCC generally shows high-grade malignancy, but no treatment policy has yet been established [11]. Surgery should thus be recommended even if the cancer is superficial [11]. Most reports have indicated that EBSCC is mainly treated with surgery [4, 12]. In terms of chemotherapy, sporadic reports have shown that chemoradiotherapy or chemotherapy can be expected to show efficacy [13–15]. The survival rate of patients with stage I or II EBSCC is considered to be similar to that of those with ESCC [4, 12]. However, the 5-year survival rate in stage III or IV AEBSCC patients is reportedly 10.5%, which indicates that AEBSCC carries a significantly poorer prognosis than the more common ESCC [4]. In Japan, the combination of preoperative chemotherapy and surgery is accepted as standard treatment for stage II or III ESCC based on findings from a Japan Clinical Oncology Group trial (JCOG9907) [16]. The 3-year survival rate of patients who did not undergo surgery for stage II or III ESCC is reportedly 45% [17]. Therefore, multimodal treatment is considered to be important for AEBSCC and some reports have stated that aggressive treatment of metastatic sites led to long-term survival [18, 19]. However, there are no reports referring to radiotherapy, according to our literature search. Our present patient has maintained recurrence-free survival for approximately 2 years since completion of radiotherapy, suggesting that radiotherapy might be effective as local treatment. There are three reports suggesting radiotherapy to be effective, although the patients were not treated with radiotherapy only. One report described a patient with 5-year survival administered radiotherapy when mediastinal lymph node metastasis appeared after surgery for stage III AEBSCC [20] (Table 1). Another report documented 4-year survival in a patient in whom the therapeutic effects on stage IVA AEBSCC were favorable, but radiotherapy was performed only at the site of local recurrence [21]. A patient with long-term survival who underwent stereotactic radiotherapy for a solitary lung metastasis was also reported [13]. However, a stage III AEBSCC patient for whom chemotherapy was performed after surgery reportedly died, 8 months later, of lung and liver metastases [22] (Table 1); the lesion site was the lower esophagus in that patient reported by Nishida et al. Other patients with upper and middle esophageal lesions had better outcomes [4]. This would explain why the aforementioned patient survived.

We evaluated nine case reports [18, 19, 22–28] describing patients with AEBSCC in the same stage (III) as that in our patient, 10 cases in total, and found the median survival time to be 13 months. Surgery was performed in all cases, but radiotherapy was performed only in three cases. Two of these three, including ours, experienced long-term survival. However, little can be inferred from so few case reports. With multimodal treatment, efficacy of pre-operative chemoradiotherapy and even chemoradiotherapy without surgery can be anticipated (Table 1).

The question of whether EBSCC is rare and accounts for approximately 10% of all esophageal cancers [10] was not discussed in previous reports. EBSCC is histologically characterized by a submucosal tumor-like growth, due to tumor nests invading the submucosal layer and deeper structures, and the formation of an elevated lesion. Therefore, patients with type I esophageal carcinoma may be diagnosed with squamous cell carcinoma based on biopsy findings only from the superficial layer of the tumor, raising the possibility of including those with basaloid carcinoma. If EBSCC is included in the category of type I advanced esophageal carcinomas, the proportion of this tumor among all esophageal cancers may well increase. In the future, if a tumor is mainly a type I advanced esophageal cancer, we should advocate biopsy in the deep portions of the tumor, considering the possibility of EBSCC.

Our clinical experience suggests that type I esophageal carcinoma is highly sensitive to radiotherapy. Accordingly, there is only one report [29] suggesting radiotherapy to exert beneficial effects on type I advanced esophageal carcinoma. EBSCC is considered to have a high metastatic potential because the tumor spreads to the submucosa. However, Thariat et al. [7] reported that patients with BSCC of the head and neck receiving irradiation did not have poorer outcomes than those with squamous cell carcinoma of the head and neck with positive lymph node status. Therefore, radiotherapy might be beneficial as a local treatment for basaloid carcinoma.

In conclusion, this is the first reported case with AEBSCC treated only with radiotherapy. This is a rare disease, but we intend to make efforts to increase the diagnostic yield. The radiosensitivity of AEBSCC needs to be further examined in future studies.

## Abbreviations

AEBSCC: Advanced esophageal basaloid squamous cell carcinoma
BSCC: Basaloid squamous cell carcinoma
EBSCC: esophageal basaloid squamous cell carcinoma
ESCC: esophageal squamous cell carcinoma
